# Understanding integrated care: a complex process, a fundamental principle

**DOI:** 10.5334/ijic.1144

**Published:** 2013-03-22

**Authors:** Nick Goodwin

**Affiliations:** Editor-in-Chief, International Journal of Integrated Care

## Understanding integrated care: a complex process, a fundamental principle

Over the past year I have been involved in a range of research and development activities that seek to understand and/or promote the successful adoption of integrated care. In each of these, a common opening statement from protagonists is to typically say that “there is no universally accepted definition of integrated care, no one model of care that can be replicated locally, and little evidence to tell us that it works”. Whilst the latter might be disputed it remains true that people struggle with what integrated care means and particularly how it can be applied.

The reason for the continued debate on the meaning and logic of integrated care is the polymorphous nature of a term that has been applied from several disciplinary and professional perspectives and one that is associated with diverse objectives [1]. At its most basic, integrated care is of course a simple idea—combining parts so that they work to form a whole (i.e., integration) in order to optimise care and treatment to people where fragmentations in care have led to a negative impact on their care experiences and outcomes. However, it is the *process* of integration to achieve better outcomes for people that is so complex and difficult to describe.

In reviewing the literature, different taxonomies of integrated care have supported this conceptualisation by variously examining (after Nolte and McKee [2]): *types* of integration (e.g., organisational, professional, functional); *breadth* of integration (e.g., vertical, horizontal, virtual); *degree* of integration (i.e., across the continuum: linkage, co-ordination to full integration); and *processes* of integration (i.e., cultural and social as well as structural and systemic). However, relatively few attempts have been made to understand the full complexity of integrated care initiatives—for example through the lens of complex adaptive systems [3] or the idea that better care co-ordination to people is the result of activities undertaken at multiple levels (e.g., systemic, organisational, professional) [4].

Recent work to develop a *Development Model for Integrated Care* has helped describe the necessary steps in the implementation process, albeit within the context of disease management programmes in the Netherlands [5]. Common to this and other conceptual models is the recognition that integrated care is a ‘complex intervention’ where management and organizational processes to support integrated care occur at many levels simultaneously. They recognise that differing local and national contexts are highly influential in how receptive a care system might be to support integrated care and that continuous adaptations occur over time. Understanding ‘what works’ within these dynamics is therefore problematic both for researchers as well as practitioners.

In this edition of *IJIC*, the article by Pim Valentijn and colleagues [6] has created a new and unifying conceptual framework through which to understand integrated care. Their approach has placed person-focused population-based care as the guiding principle for achieving integration across the care continuum, with different integration processes playing inter-connected roles on the macro- (system integration), meso- (organisational, professional) and micro-level (clinical, service and personal integration). Functional integration (e.g., communication and IT) and normative integration (e.g., shared cultural values) ensure connectivity between the various levels. The final framework is both elegant and useful as a way of conceptualising the inter-relationships among the different dimensions of integrated care, and should also be of help to support research analysis where this seeks to understand integrated care’s complexity.

The other important insight that Valentijn et al. [6] bring forward is the similarity between the overarching objectives of integrated care with that of primary care—for example, in terms of promoting co-ordination and continuity of care, equity of access and public health. This leads to the recognition that integrated care as a concept should be seen as so much more than the sum of a range of organisational processes acting at different levels. As with primary care, integrated care should rank alongside universal health coverage and equity of access as a core property of high-quality health systems since, without it, care experiences and outcomes are unlikely to be as good as they should be. So whilst it is important to better comprehend the complex and multi-dimensional nature of integrated care as a process, it is also important to recognise that integrated care is a fundamental design principle.
